# Review Article: The acceptability and effectiveness of standardised diagnostic assessment approaches in children and young people's mental health services – an updated systematic review

**DOI:** 10.1111/camh.70007

**Published:** 2025-06-27

**Authors:** Salah Basheer, Sue Fen Tan, Ranjitha David, Pallab Majumder, Kapil Sayal

**Affiliations:** ^1^ Institute of Mental Health, School of Medicine, Jubilee Campus University of Nottingham Nottingham UK; ^2^ Nottinghamshire Healthcare NHS Foundation Trust Nottingham UK

**Keywords:** Standardised diagnostic assessments, child and adolescent mental health, utility, attitudes, acceptability, effectiveness

## Abstract

**Background:**

Standardised diagnostic assessment (SDA) tools are designed to comprehensively evaluate various mental health disorders and support accurate diagnoses. While evidence for their routine use in child and adolescent mental health services (CAMHS) remains limited, increasing service demands underscore the need for efficient clinical assessments. This updated review examines the acceptability and clinical utility of SDA tools in CAMHS.

**Method:**

A systematic review of 464 studies published between January 2013 and February 2025 was conducted (PROSPERO: CRD42024494051). Ten studies met the inclusion criteria, comprising eight cross‐sectional studies and two randomised controlled trials.

**Results:**

SDA tools reviewed included Development and Wellbeing Assessment (DAWBA), Mini‐International Neuropsychiatric Interview for Children and Adolescents (MINI‐KID) and Kiddie Schedule for Affective Disorders and Schizophrenia (KSADS). DAWBA improved referral decision accuracy, while both DAWBA and MINI‐KID had acceptable agreements with expert consensus diagnoses. MINI‐KID and KSADS were able to identify more cases than routine clinical assessments. However, DAWBA did not show effectiveness in changing diagnostic practice. Clinicians were favourable of SDA tools' psychometric properties but less favourable of diagnostic labels and feasibility in practice. Psychiatrists and psychologists were more likely than other healthcare professionals to use these tools in practice.

**Conclusions:**

This review highlights that while SDA tools may aid referral decisions, evidence supporting their overall effectiveness in CAMHS is lacking. Clinician concerns pose barriers to routine use, requiring further research before implementation.

**Key Practitioner Message**

- What is known?
  A previous systematic review demonstrated mixed findings regarding the utility of SDA tools as adjuncts to routine CAMHS practice, with some evidence for improving the detection of emotional disorders and other comorbidities.
- What is new?
  This updated review highlights that SDA tools could improve referral decision‐making. However, current evidence does not support the overall effectiveness of implementing SDA tools in CAMHS.
- What is significant for clinical practice?
  Broader clinician concerns about the utility of diagnostic labels and feasibility of SDA tools needs to be addressed.

## Introduction

The complex nature of mental health disorders in children and young people (CYP) presents significant diagnostic challenges in child and adolescent mental health services (CAMHS) ( O'Connor, Downs, Shetty, & McNicholas, 2020). The multidisciplinary nature of CAMHS is another potential barrier to diagnosis assignment, as professionals from diverse training backgrounds may conceptualise CYP's difficulties differently and hold varying opinions around labelling these difficulties with a specific diagnosis (Jones & Larner, 2004; Michelson et al., 2011). Varying degrees of certainty in diagnostic language among clinicians complicate this issue (O'Connor, Downs, McNicholas, et al., 2020), particularly since many treatment guidelines for mental health disorders rely on categorical diagnoses. Furthermore, there is also increasing demand and waiting times for CAMHS and incomplete clinical information contributes significantly to referral rejections (Frith, 2017).

Implementing standardised diagnostic assessment (SDA) tools could mitigate some of these problems and potentially improve long waiting times and efficiency in CAMHS, especially if used before clinical appointments (Martin, Fishman, Baxter, & Ford, 2011). When used as adjuncts, SDA tools could support and enhance clinical assessments. This is because SDA tools are comprehensive tools that assess a broad range of mental health disorders in a holistic manner using single or multiple informants and assign symptoms to a diagnostic category according to established diagnostic manuals like the diagnostic and statistical manual (DSM) or the International Classification of Diseases (ICD) (Reeves, Charter, & Ford, 2016). Examples of SDA tools include the Development and Wellbeing Assessment (DAWBA), Mini International Neuropsychiatric Interview for Children and Adolescents (MINI‐KID) and Kaufman Schedule for Affective Disorders and Schizophrenia (KSADS) (Goodman, Ford, Richards, Gatward, & Meltzer, 2000; Kaufman et al., 1997; Sheehan et al., 2010).

While SDA tools could serve as useful guides to support evidence‐based practice, their application in clinical settings remains inconsistent (Jensen‐Doss, 2015). In the UK, National Institute for Health and Care Excellence (NICE, 2019) recommends using SDA tools as potential aids in detecting depression in CYP (NICE, 2019), but no other national guidelines, to our knowledge, recommend their routine use in clinical practice. A systematic review by Reeves et al. (2016) demonstrated mixed findings regarding the utility of SDA tools, with some evidence for improving the detection of emotional disorders and other comorbidities (Reeves et al., 2016). This systematic review included studies published up to 2013, and more studies have emerged in this field over the past decade. Therefore, it is imperative to conduct an updated systematic review focusing on recent studies examining clinicians' attitudes towards SDA tools and summarising new evidence regarding the utility of SDA tools in CAMHS. The questions that this review aims to assess are:

- Are SDA tools acceptable to various stakeholders, including young people, parents/carers and healthcare professionals?
- Are SDA tools effective in the clinical management of mental health disorders in children and young people up to the age of 18 years?

## Methods

The search strategy was designed to emulate the systematic review by Reeves et al. (2016). The databases were searched for articles published from January 2013 till 3rd of February 2025. The search terms covered the following themes: child/adolescents, disorders, diagnostic assessments and mental health services (see Appendix S1 for the details of search). The following databases were searched:

- PubMed
- ProQuest ASSIA
- Cochrane Library
- DARE
- Ovid Embase
- ProQuest International Bibliography of Social Sciences
- Ovid MEDLINE® ALL
- ProQuest PsycINFO

The systematic review was registered with the PROSPERO database on 17 January 2024 (CRD42024494051). Duplicate studies were removed using the Rayyan software. Two authors (SB and SFT) independently screened the titles and abstracts, resolving any disagreements through face‐to‐face discussions. The same co‐authors independently reviewed the full‐text articles to determine their eligibility.

Studies were included if they evaluated the acceptability or effectiveness of SDA tools in CYP under 18 years old attending a mental health care clinic and the SDA tools provided categorical diagnoses for a wide range of mental health disorders. Like Reeves et al. (2016), studies focused solely on diagnostic assessments for a single specific disorder (like Body Dysmorphic Disorder) were excluded, as this review aimed to examine the evidence on broad‐based diagnostic assessments. However, this exclusion did not extend to assessment of a category or a group of disorders (like emotional disorders). We also excluded studies that involved the use of multiple brief dimensional measures, as this approach differs fundamentally from SDA tools designed to assign categorical diagnoses. Further, we excluded case series reports, conference papers, posters, meeting abstracts, unpublished literature and non‐English articles.

For data synthesis, SB and SFT extracted data from full‐text articles into a tabulated form, analysed the data, identified themes in the extracted data and populated the findings into a structured table. The quality of studies was assessed independently by SB and SFT using the Joanna–Briggs Institute (JBI) checklist (Munn et al., 2020). No studies were excluded after the quality appraisal process. Due to the methodological diversity of the included studies, meta‐analysis was not feasible.

## Results

### Study details

**Figure 1:**
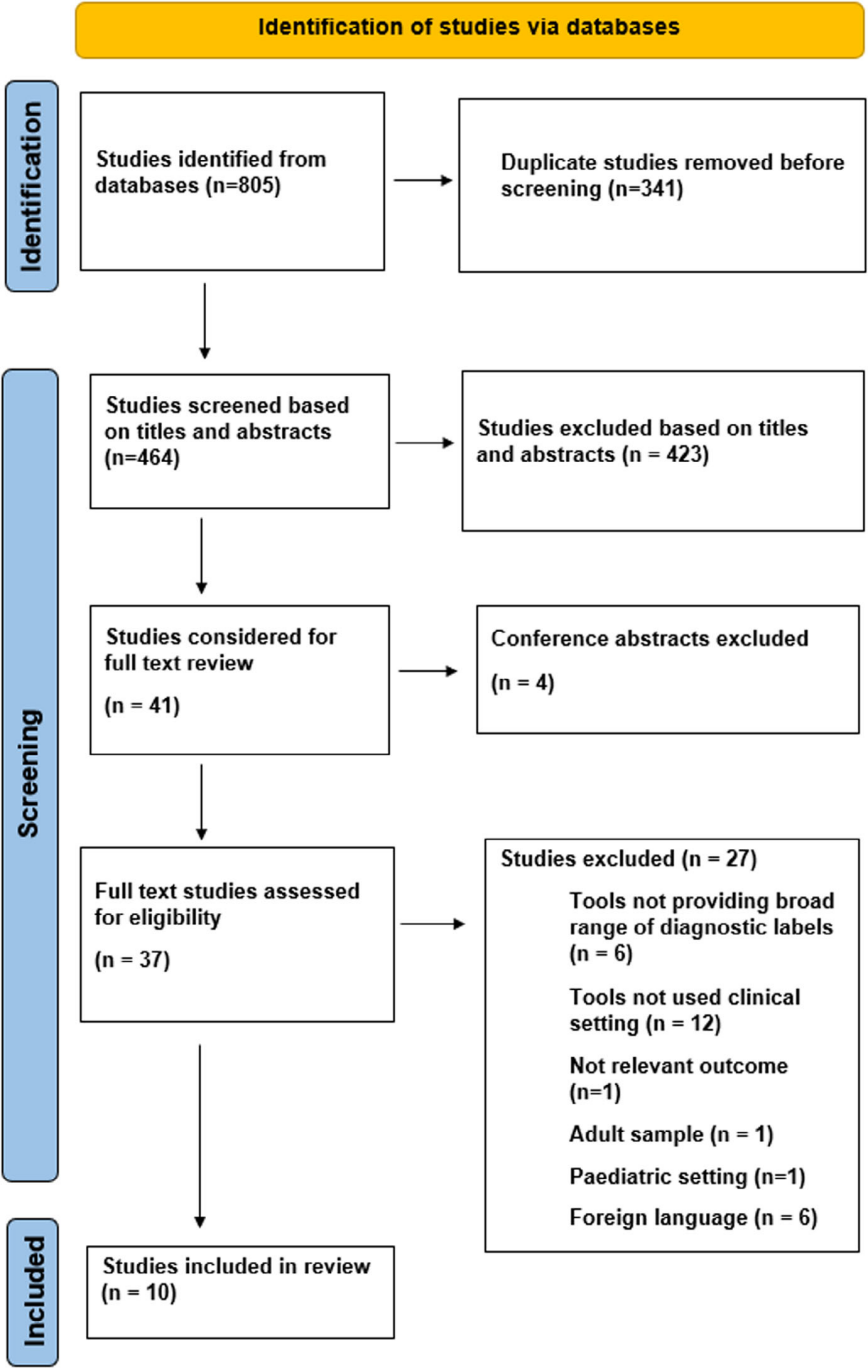
Screening process for eligible studies

Figure 1 illustrates the screening process and selection based on PRISMA guidelines. The search identified 10 articles. One article (Sayal et al., 2025) is a recently published randomised controlled trial (RCT) which had not yet been indexed in the search databases was included based on ‘expert knowledge’ that exists. Most studies were from Europe (three from Norway, two from Sweden and one each from United Kingdom, Netherlands and Denmark) and two from the United States. Seven studies investigated diagnostic practice when SDA tools were used in clinical practice (Table 1). Five were cross‐sectional studies (Aydin et al., 2022; Brøndbo et al., 2013; Hansen et al., 2016; Högberg et al., 2019; Jensen‐Doss et al., 2014) and two were randomised controlled trials (Hansen et al., 2023; Sayal et al., 2025). Four studies explored the use of DAWBA (Aydin et al., 2022; Brøndbo et al., 2013; Hansen et al., 2023; Sayal et al., 2025) while two used KSADS‐PL (Hansen et al., 2016; Jensen‐Doss et al., 2014). One study explored the MINI‐KID (Högberg et al., 2019). In addition, three cross‐sectional surveys explored clinician attitudes towards SDA tools (Table 2) (Bjaastad et al., 2019; Cook et al., 2017; Danielson et al., 2019).

**Table 1:** Overview of studies that looked at referrals and diagnostic processes using standardised diagnostic assessment tools

Authors	Aydin et al. (2022)	Brøndbo, Mathiassen, Martinussen, Handegård, and Kvernmo (2013)	Hansen et al. (2016)	Hansen, Kjaersdam Telléus, Mohr‐Jensen, Færk, and Lauritsen (2023)	Högberg et al. (2019)	Jensen‐Doss, Youngstrom, Youngstrom, Feeny, and Findling (2014)	Sayal et al. (2025)
Setting/Country	CAMHS centre in Netherlands	Urban university or semi‐rural CAMHS clinics in North Norway	CAMHS outpatient clinics in Norway	CAMHS Department of the North Denmark Region	CAMHS outpatient clinics in Sweden	Community mental health centre in United States	CAMHS outpatient clinics in United Kingdom
Study design	Cross‐sectional study	Cross‐sectional study	Cross‐sectional study	Randomised feasibility trial	Cross‐sectional study	Cross‐sectional study	Randomised controlled trial
SDA tool used	DAWBA	DAWBA	KSADS‐PL	DAWBA	MINI‐KID	KSADS	DAWBA
Diagnoses assessed	Anxiety, depression, ADHD, ASD, behavioural disorders	Separation anxiety, specific phobias, social phobia, panic attacks, agoraphobia, PTSD, generalised anxiety, OCD, depression, deliberate self‐harm, hyperkinetic disorder, conduct disorder, developmental disorders, eating difficulties	Non OCD anxiety disorders: for example, panic disorder, separation anxiety, social phobia, specific phobia, generalised anxiety disorder, PTSD	Affective disorders, anxiety disorders, eating disorders, ADHD, ASD, tic disorders	Depressive disorders, suicidality, bipolar disorders, anxiety disorders, ADHD, PTSD, OCD, alcohol abuse, substance abuse, tic disorders, disruptive disorders, psychotic disorders, stress disorders, eating disorders, pervasive developmental disorders	Depression, ADHD, PTSD, bipolar disorders, specific phobia, OCD, generalised anxiety disorder, separation anxiety disorder, selective mutism, adjustment disorder with anxiety, disruptive behaviour, elimination disorders	Separation anxiety disorder, specific phobia, social phobia, panic disorder, agoraphobia, generalised anxiety disorder, PTSD, OCD, depression, oppositional defiant disorder, conduct disorder
Sample size (*N*) and participant type	1259 young people (5–18 years old)	286 young people (5–18 years old)	407 young people (7–13 years old)	160 young people (6–17 years old)	101 young people (4–18 years old)	391 young people (6–18 years old)	615 (of 1225 total sample) (5–17 years old)
Relevant outcomes	Predictive value of DAWBA‐assigned diagnoses against expert consensus diagnoses	Diagnostic agreement between DAWBA‐assigned diagnoses and routine clinical diagnoses	Proportion of non‐OCD anxiety disorders diagnosed in childhood using KSADS‐PL in clinics in comparison to the proportion of similar diagnoses in the Norwegian Patient Register	Proportion of referred children that received correct referral decisions and fulfilled criteria for CAMHS assessment when DAWBA was used an adjunct to referral letters and SDQ	Diagnostic agreement between MINI‐KID‐assigned diagnoses, routine clinical diagnoses and expert consensus diagnoses	Diagnostic agreement between KSADS‐informed expert consensus diagnoses and routine clinical diagnoses	Proportion of emotional disorders diagnosed, CAMHS referral acceptance and CAMHS intervention offered at 12 & 18 months when DAWBA was used as an adjunct to routine referral letters and clinical assessment. Cost effectiveness of DAWBA
Main findings	DAWBA had equal sensitivity and specificity in predicting diagnoses and was useful in diagnoses assignment	Fair diagnostic agreement between DAWBA‐assigned diagnoses and routine clinical diagnoses (kappa = 0.41–0.49; overall percentage diagnostic agreement for any disorder = 74%, emotional disorder = 74%, hyperkinetic disorder = 81%, conduct disorder = 79%, other disorders = 90%, comorbid disorder = 80%)	32.7% met the criteria for a non‐OCD anxiety disorder when KSADS‐PL was used compared with 5% of non‐OCD anxiety disorders recorded in the Norwegian Patient Register	DAWBA as an adjunct to referral letters demonstrated higher sensitivity (0.83 vs. 0.63) and specificity (0.42 vs. 0.30) in terms of referral decision accuracy than SDQ	MINI‐KID provided additional preliminary diagnoses compared with routine clinical practice There was an overall acceptable percentage agreement (79.5%) between MINI‐KID‐assigned diagnoses and expert consensus diagnoses (overall percentage diagnostic agreement for any disorder = 76.2%, any anxiety disorder = 70.3%, ADHD = 74.3%, disruptive disorder = 76.2%, depressive disorders = 71.7%, OCD = 91.1%, tic disorder = 87.8%, pervasive developmental disorder = 85.2%)	Lower diagnostic agreement (kappa < 0.4) except for depressive disorder (kappa = 0.43) and PTSD (kappa = 0.46) between KSADS‐informed expert consensus diagnoses and routine clinical diagnoses (positive percentage diagnostic agreement for depression = 15%, bipolar disorders = 4%, anxiety disorders = 2%, ADHD = 37%, disruptive behaviour = 44%, elimination disorders = 4%, PTSD = 8%)	No difference between DAWBA and routine clinical assessment groups for emotional disorder diagnoses (11% vs. 12%), CAMHS referral acceptance (57% vs. 55%), and CAMHS intervention offered (42% vs. 39%); DAWBA was not more cost‐effective than routine clinical assessment; 80% of participants completed the DAWBA with almost all of them completing it online

ADHD, attention‐deficit hyperactivity disorder; ASD, autism spectrum disorder; CAMHS, child and adolescent mental health services; DAWBA, Development and Wellbeing Assessment; KSADS, Kaufman Schedule for Affective Disorders and Schizophrenia; KSADS‐PL, Kaufman Schedule for Affective Disorders and Schizophrenia, present and lifetime version – parents only; MINI‐KID, Mini International Neuropsychiatric Interview for Children and Adolescents; OCD, obsessive‐compulsive disorder; PTSD, post‐traumatic stress disorder; SDQ, Strengths and Difficulties Questionnaire.

**Table 2:** Overview of studies on clinicians' attitudes towards standardised diagnostic assessment tools

Authors	Bjaastad et al. (2019)	Cook, Hausman, Jensen‐Doss, and Hawley (2017)	Danielson, Månsdotter, Fransson, Dalsgaard, and Larsson (2019)
Setting/Country	Norway	United States	Sweden
Study design	ASA survey	National survey	ASA survey
SDA tool assessed	Not specified	CBCL, CGAS, KSADS, Folstein Mini‐Mental Status Examination, Dyadic Parent–Child Interaction Coding System II, Child and Adolescent Functional Assessment Scale	Not specified
Sample size (*N*) and participant type	606 clinicians	1510 clinicians	345 clinicians
Outcomes	Clinicians' attitudes towards SDA tools, predictors of SDA tool use and differences in attitudes towards SDA tools by clinical profession	Overall use of SDA tools in clinical practice and differences in attitudes towards SDA tools by clinical profession	Clinicians' attitudes towards SDA tools and differences in attitudes towards SDA tools by clinical profession
Main findings	Clinicians felt most positively regarding SDA tools' psychometric properties, clinical feasibility and benefits over clinical judgement alone and these predicted the use of SDA tools; CAMHS clinicians had significantly more positive attitudes towards SDA tools than adult mental health clinicians; Medical doctors and psychologists had more positive attitudes towards SDA tools	SDA tools were used less frequently than non‐SDA tools; Psychologists and psychiatrists used SDA tools more frequently; The adequacy of clinicians' prior training strongly predicted the use of SDA tools	Clinicians felt most positively regarding SDA psychometric quality and were less positive to utility of diagnosis, clinical feasibility and benefits over clinical judgement; Counsellors were least positive towards SDA tools

ASA, Attitudes towards Standardised Assessment Scales; CAMHS, child and adolescent mental health service; CBCL, Child Behaviour Checklist; CGAS, Children's Global Assessment Scale; KSADS, Schedule for Affective Disorders and Schizophrenia for School‐Age Children.

### Effectiveness of SDA tools in referral and diagnostic processes

Table 1 summarises seven studies evaluating the effectiveness of various SDA tools in CAMHS, focusing on referral and diagnostic processes. When used as an adjunct to referral letters, DAWBA improved referral decision accuracy by ensuring that referrals of CYP with true clinical needs were more likely to be accepted by CAMHS (Hansen et al., 2023), but overall referral acceptance rates were not significantly higher than if referral information was used alone (Sayal et al., 2025). DAWBA demonstrated higher and more balanced sensitivity and specificity in referral and diagnostic decisions compared with standalone referral letters and non‐SDA tools like the Strengths and Difficulties Questionnaire (SDQ) (Aydin et al., 2022; Hansen et al., 2023). Further, online DAWBA‐assigned diagnoses showed fair agreement with routine clinical diagnoses (Brøndbo et al., 2013). Though 80% of the participants offered completed DAWBA online, its integration into routine clinical practice did not significantly affect the clinical diagnosis of emotional disorders, time to diagnosis, or receipt of treatment (Sayal et al., 2025). Additionally, the addition of the DAWBA was not found to be more cost‐effective than usual care (Sayal et al., 2025).

MINI‐KID was able to independently identify more diagnoses than routine initial clinical assessments, particularly for anxiety, obsessive‐compulsive disorders (OCD), tics and behavioural disorders (Högberg et al., 2019). It showed acceptable overall agreement with expert consensus and routine clinical diagnoses and had high agreement for OCD, tics and pervasive developmental disorders (Högberg et al., 2019). Additionally, MINI‐KID required less time to administer, averaging 46 min compared with the 60 min typically needed for routine assessments.

KSADS‐PL identified CYP who met the DSM‐IV criteria for non‐OCD anxiety disorders (panic disorder/agoraphobia, separation anxiety, social phobia, specific phobia, generalised anxiety disorder and post‐traumatic stress disorder) approximately six times more frequently compared with routine clinical assessment (Hansen, Oerbeck, Skirbekk, & Kristensen, 2016). Notably, half of these CYP did not present with anxiety as a referral symptom (Hansen et al., 2016). However, overall agreement between routine clinical diagnoses and expert consensus diagnoses incorporating KSADS was lower (kappa < 0.4), except for depressive disorder and post‐traumatic stress disorder (PTSD), which were attributed both to missed diagnoses and additional incorrect diagnoses (Jensen‐Doss et al., 2014). Diagnostic agreement improved when CYP had clearer clinical presentations (high or low symptom severity), fewer comorbidities, were older, or had better family functioning.

### Clinicians' attitudes to SDA tools

A US national survey involving 1510 clinicians revealed that a large proportion of clinicians (76.7%) did not use SDA tools in their assessments (Cook et al., 2017). All three studies assessing attitudes found that SDA tools were more acceptable and widely used by psychologists and psychiatrists compared with other mental health professionals (Bjaastad et al., 2019; Cook et al., 2017; Danielson et al., 2019) including counsellors with degrees in social work or psychotherapy, nurses and individuals with other professional backgrounds, such as mental health support workers. Findings from cross‐sectional surveys of Norwegian and Swedish clinicians regarding their attitudes towards SDA tools showed that while clinicians expressed predominantly positive sentiments regarding their psychometric properties, their views on utility of diagnostic labels, feasibility in routine clinical assessment and benefits over clinical judgement were less favourable (Bjaastad et al., 2019; Danielson et al., 2019). However, CAMHS clinicians were more likely than adult counterparts to use SDA tools to screen for diagnoses and were more favourable with regards SDA tools being reliable, valid and feasible in clinical practice (Bjaastad et al., 2019). The adequacy of training in SDA tools, type of profession and clinicians' opinion of diagnostic labels, SDA tools' psychometric properties, feasibility of use and benefit over clinical judgement predicted the use of SDA tools in clinical practice (Bjaastad et al., 2019; Cook et al., 2017; Danielson et al., 2019). There were no differences in attitudes towards SDA tools between Norwegian and US clinicians (Bjaastad et al., 2019). However, Swedish clinicians were more positive than US clinicians regarding utility of diagnostic labels and SDA tool's benefit over clinical judgement (Danielson et al., 2019).

### Quality appraisal

Due to the heterogeneity of included studies, the JBI cross‐sectional and randomised RCT quality appraisal tools were used. Table 3 provides a summary of the quality appraisal for all cross‐sectional studies in this review and Table 4 provides the summary for randomised trials. Overall, the studies had clear inclusion criteria, detailed participant information and study settings. Appropriate statistical analyses were performed for all studies. In studies examining the effectiveness of SDA tools in referral and diagnostic processes, DAWBA, MINI‐KID and KSADS‐PL demonstrated good validity and reliability. The ASA (Attitudes towards Standardised Assessment) scale that studied clinicians' attitudes towards SDA tools had good psychometric properties. In studies that compared SDA‐based diagnostic classification, Aydin et al. (2022) and Högberg et al. (2019) used expert consensus diagnosis while Brøndbo et al. (2013) and Jensen‐Doss et al. (2014) used routine clinical assessment. Cook et al. (2017) and Bjaastad et al. (2019) excluded clinicians who do not regularly use SDA tools from their surveys, and this meant that reasons for lack of use of SDA tools could not be explored, leading to non‐response bias. There were differences in SDA tool administration (Högberg et al., 2019) and the number and sources of informants (Brøndbo et al., 2013) in some studies. Both the randomised trials demonstrated good internal validity overall. However, limitations included the non‐blinding of participants in both studies, accidental unblinding of some clinicians in the feasibility trial (Hansen et al., 2023), and the lack of blinding for outcome assessors in the RCT (Sayal et al., 2025).

**Table 3:** Quality appraisal of cross‐sectional studies in the review based on the Joanna–Briggs Institute cross‐sectional checklist

Authors	Aydin et al. (2022)	Högberg et al. (2019)	Hansen et al. (2016)	Jensen‐Doss et al. (2014)	Brøndbo et al. (2013)	Cook et al. (2017)	Bjaastad et al. (2019)	Danielson et al. (2019)
Inclusion criteria clarity	Yes	Yes	Yes	Yes	Yes	Yes	Yes	Yes
Detailed study subjects and the setting information	Yes	Yes	Yes	Yes	Yes	Yes	Yes	Yes
Standard criteria for measurement of condition	Yes	Yes	Yes	N/A	N/A	Yes	Yes	Yes
Confounding factors identified	No	Yes—interviewers could opt out of follow‐up MINI‐KID questions, some medical records had insufficient information or had diagnoses not present in MINI‐KID	N/A	No	Yes—different number of DAWBA informants and clinicians' subject in assigning diagnoses based on expertise and duration of assessment	Yes—exclusion of clinicians who do not regularly use SDA tools but reasons for lack of use not explored, recall bias in self‐reports, clinicians were recruited only from large professional organisations	Yes—exclusion of clinicians who do not regularly use SDA tools but reasons for lack of use not explored	No
Confounding factors controlled for	N/A	No	N/A	N/A	Partial—blinding for clinicians assigned to DAWBA group and routine group	No	No	N/A
Appropriate statistical analysis used	Yes	Yes	Yes	Yes	Yes	Yes	Yes	Yes

DAWBA, Development and Wellbeing Assessment; MINI‐KID, Mini International Neuropsychiatric Interview for Children and Adolescents.

**Table 4:** Quality appraisal of randomised controlled studies in the review based on the Joanna–Briggs Institute randomised controlled checklist

Authors	Hansen et al. (2023)	Sayal et al. (2025)
Internal validity		
Bias related to selection and allocation	Randomised but concealment of allocation was not possible due to the nature of the study	Randomised but concealment of allocation was not possible due to the nature of the study
Bias related to administration of intervention	Not possible to assess due to the nature of the study	Not possible to assess due to the nature of the study
Bias related to assessment, detection and measurement of outcome	Yes, except in some cases where participants revealed their allocation	Not possible to blind site researchers, clinicians and some trial staff, except the adjudication committee and trial statisticians due to the nature of the study
Bias related to participant retention	No	No
Statistical conclusion validity		
Analysis of participants in groups that they were randomised	Yes	Yes
Appropriate statistical analysis used	Yes	Yes
Appropriate trial design	Yes	Yes

## Discussion

This updated review focused on studies (published between 2013 and 2025) that used SDA tools in CAMHS outpatient settings to explore current acceptability and effectiveness in clinical practice, focusing on findings pertinent to clinical services such as referral decisions, diagnostic practice and clinician attitudes.

This review highlighted significant variability in diagnostic assignment in CAMHS outpatient settings (Brøndbo et al., 2013; Hansen et al., 2016; Högberg et al., 2019; Jensen‐Doss et al., 2014). Missed diagnoses in clinical practice were attributed to the presence of comorbid mental health disorders or unclear clinical presentations (Hansen et al., 2016; Jensen‐Doss et al., 2014). Implementation of routine SDA tools in clinical practice may mitigate missed diagnosis and improve diagnostic accuracy as SDA tools contain questions pertaining to DSM and ICD disorders. This was evident in the study by Hansen et al. (2016) in which anxiety disorder was diagnosed even in the absence of anxiety symptoms in the referral letter. A structured and standardised clinical assessment with SDA tools could decrease the likelihood of missing comorbid mental health disorders, irrespective of the administrating clinician. This is because there is good interrater reliability for SDA tools such as DAWBA and MINI‐KID for most mental health disorders (Aebi et al., 2012; Sheehan et al., 2010). Furthermore, clinicians may not necessarily cover symptoms of every disorder in a routine clinical assessment due to time constraints. Högberg et al. (2019) demonstrated that SDA tools may be able to save clinicians time during assessments and albeit only a few minutes, this could have a cumulative effect.

Previously, DAWBA was shown to be useful in detecting emotional disorders when utilised along with clinical assessments (Aebi et al., 2012; Ford et al., 2013) and accurately diagnosing ADHD if used by senior clinicians (Foreman, Morton, & Ford, 2009). However, no impact of DAWBA in changing diagnostic practice was demonstrated in the recent, larger RCT (Sayal et al., 2025). Like Ford et al. (2013), Sayal et al. (2025) also found no effect of DAWBA on the treatment provided. Several factors may have influenced these findings, including how clinicians utilised the reports generated by SDA tools. Additionally, Sayal et al. (2025) reported a low overall clinical diagnostic rate, despite DAWBA indicating a high likelihood of diagnosis. This suggests potential differences in attitudes among the mental health professionals towards clinical diagnosis in general. Despite incorporating more studies (cross‐sectional studies and RCTs) than the previous review (Reeves et al., 2016), this review does not find evidence to support the overall effectiveness of SDA tools in improving diagnostic practice in routine child mental health clinics.

Accuracy in referral decision‐making is crucial to ensure quality care is being delivered efficiently without unnecessary resource expenditure. Referral acceptance to CAMHS necessitates the presence of symptoms suggestive of a mental health disorder that is too severe to be managed in primary care. Our review showed that DAWBA, when used as an adjunct to referral letters, improved the accuracy of CAMHS referral decisions (Hansen et al., 2023). However, the use of DAWBA did not increase referral acceptance rates or reduce the time to diagnosis (Sayal et al., 2025). This suggests that while SDA tools could potentially enhance the quality of referral information and aid in screening referrals, they may not be inherently time or cost‐saving in clinical practice, especially if it is unclear whether clinician utilise additional information from DAWBA in their assessments (Sayal et al., 2025). This is relevant given the increasing demand for CAMHS services; in England, for example, the number of CYP referred to CAMHS nearly doubled between 2019 and 2023 and is projected to continue rising (NHS Digital, 2023). While most of the CAMHS referrals originate from primary care, CYP are also referred from emergency services, educational and social care settings and other secondary services, including paediatrics (NHS Digital, 2023). DAWBA was well‐accepted by 98% of parents of CYP in paediatric neurology outpatient clinics and could identify at least one DSM‐IV disorder in 60%–70% of CYP without requiring additional time or intervention from paediatricians (Bennett et al., 2019). This underlines the potential of using SDA tools in other secondary healthcare settings as adjunct referral tools, but further evaluation is needed for their role in standard referral pathways given their limited impact on CAMHS service‐related outcomes.

Further, our review identified numerous challenges in embedding SDA tools routinely within CAMHS. CAMHS comprises professionals from various disciplines with different training and attitudes towards SDA tools. Psychiatrists and psychologists had more positive attitudes towards SDA tools, possibly because their training is more attuned to diagnostic considerations (Bjaastad et al., 2019; Cook et al., 2017; Danielson et al., 2019). This finding is consistent with two studies in the previous review that highlighted how the training background of professionals influences their use of SDA tools in clinical practice (Jensen‐Doss & Hawley, 2011; Martin et al., 2011). Moreover, while training can temporarily improve attitudes and use of SDA tools, these effects are often short‐lived (Lyon, Dorsey, Pullmann, Silbaugh‐Cowdin, & Berliner, 2015), since this finding can reflect the culture of receptiveness or reluctance around utilisation of diagnostic categories, further complicating efforts to implement SDA tools routinely and consistently in clinical settings. The previous review also found that while clinicians appreciated the standardisation and streamlining of assessments provided by SDA tools, they felt that the burden placed on families to complete these tools was a drawback (Reeves et al., 2016). Though, Sayal et al. (2025), showed high adherence of participants completing DAWBA online, further research is needed to explore both patient and family acceptability of SDA tools in CAMHS outpatient settings.

### Limitations

This review had some similar methodological challenges as the previous systematic review by Reeves et al. (2016). The search results were limited as the review was aimed at studies that only used SDA tools that cover a broad range of mental health disorders in CAMHS outpatient clinics. However, this review did not explore other assessment approaches such as transdiagnostic approaches. The studies in this review were heterogenous, and only three studies used longitudinal expert consensus data to provide estimate diagnoses (Aydin et al., 2022; Högberg et al., 2019; Jensen‐Doss et al., 2014). Previously, challenges of using different SDA tools in various settings have been explored (Angold et al., 2012). However, due to differences in study designs and SDA tools used in the included papers, it was difficult to compare the effectiveness of specific SDA tools directly or conduct a meta‐analysis. Furthermore, we only found studies from the USA and Europe, which restrict the international generalisability of our findings. We also did not find studies that specifically explored patient acceptability.

### Future directions

Before considering the routine implementation of SDA tools in CAMHS, more work is needed to establish their effectiveness for different service‐related outcomes, which to date could not be established conclusively. Much more robust work also needs to be carried out to explore the implementation challenges in routine practice looking at potential barriers such as clinician attitudes, awareness and training. Moreover, there is a lack of studies on CYP or parent acceptability of SDA tools, and without engagement from all stakeholders, implementation may not be feasible. Digital solutions, such as online versions of SDA tools, may help address clinicians' concerns around the feasibility of using SDA tools. Online versions could be quicker and easier to complete than paper versions and provide automatically generated probability bands for diagnoses of various mental health disorders (Day et al., 2022; Goodman et al., 2000; Goodman, Heiervang, Collishaw, & Goodman, 2011; Townsend et al., 2020).

## Conclusion

This review found insufficient evidence to support the routine implementation of SDA tools in CAMHS. While SDA tools may improve referral decision‐making, low receptiveness from professionals other than psychiatrists or psychologists hinders their widespread adoption in CAMHS. For SDA tools to be integrated into CAMHS, clinician concerns regarding their acceptability, utility and feasibility, alongside the involvement of CYP and families, needs to be addressed first.

## Conflict of interest statement

KS is the lead investigator for a randomised control trial investigating the effectiveness of the standardised diagnostic assessment tool in CAMHS funded by the National Institute for Health and Care Research (NIHR), Health Technology Assessment Programme (HTA) (Project Ref. 16/96/09). All other authors have declared that they have no competing or potential conflicts of interest.

## Funding information

No external funding from funding agencies in the public, commercial, or not‐for‐profit sectors was provided for this review.

## Ethics statement

This review did not require any ethical approval as it did not involve any trials involving human or animal participants.

## Supporting information


**Appendix S1** Search strategies with databases and search terms used.

## Data Availability

All data used in this systematic review are derived from publicly available sources, including published journal articles and relevant databases. The datasets supporting the findings of this review can be accessed through the respective sources cited within the manuscript. For further information regarding data sources or to request additional details, please contact the corresponding author.
