# Supplementary material for: Review Article: The acceptability and effectiveness of standardised diagnostic assessment approaches in children and young people's mental health services – an updated systematic review
Source: Child Adolesc Ment Health. 2025 Jun 27;30(4):364–74. doi: 10.1111/camh.70007 (PMC12573065; doi:10.1111/camh.70007)
Supplement: Supplementary file 1 — Appendix S1 Search strategies with databases and search terms used. [file CAMH-30-364-s001.docx]

**Appendix S1: Search strategies with databases and search terms used**

| **Platform and database**: PubMed | | |
| --- | --- | --- |
| 1 | "Child"[Mesh] OR "Minors"[Mesh] OR "Child, Preschool"[Mesh] OR "Adolescent"[Mesh] OR "Pediatrics"[Mesh] | 3534020 |
| 2 | adolescen*[Title/Abstract] OR child*[Title/Abstract] OR infant*[Title/Abstract] OR juvenile*[Title/Abstract] OR minor*[Title/Abstract] OR paediatric*[Title/Abstract] OR pediatric*[Title/Abstract] OR teen*[Title/Abstract] OR "young person*"[Title/Abstract] OR "young people"[Title/Abstract] OR youth*[Title/Abstract] | 3039466 |
| 3 | #1 or #2 | 4901901 |
| 4 | "Anxiety"[Mesh] OR "Anxiety Disorders"[Mesh] OR "Depression"[Mesh] OR "Hypochondriasis"[Mesh] OR "Dysthymic Disorder"[Mesh] OR "Stress Disorders, Post-Traumatic"[Mesh] OR "Agoraphobia"[Mesh] OR "Phobia, Social"[Mesh] OR "Obsessive-Compulsive Disorder"[Mesh] OR "Mood Disorders"[Mesh] OR "Phobic Disorders"[Mesh] | 491219 |
| 5 | agitat*[Title/Abstract] OR anger[Title/Abstract] OR anxiet*[Title/Abstract] OR anxious[Title/Abstract] OR "body dysmorphi*"[Title/Abstract] OR compulsion*[Title/Abstract] OR depress*[Title/Abstract] OR distress[Title/Abstract] OR dysthymia[Title/Abstract] OR "dysthymic disorder*"[Title/Abstract] OR flashback*[Title/Abstract] OR GAD[Title/Abstract] OR hypervigilance[Title/Abstract] OR insomnia[Title/Abstract] OR irritable[Title/Abstract] OR "lack of motivation"[Title/Abstract] OR "low mood"[Title/Abstract] OR "low motivation"[Title/Abstract] OR "low self-esteem"[Title/Abstract] OR "low self esteem"[Title/Abstract] OR "mood affective disorder*"[Title/Abstract] OR "mood disorder*"[Title/Abstract] OR "mood swing*"[Title/Abstract] OR nightmare*[Title/Abstract] OR panic[Title/Abstract] OR phobi*[Title/Abstract] OR "self-harm"[Title/Abstract] OR "self harm"[Title/Abstract] OR "selfharm"[Title/Abstract] OR "self-injur*"[Title/Abstract] OR "self injur*"[Title/Abstract] OR suicid*[Title/Abstract] OR tired*[Title/Abstract] OR withdrawn[Title/Abstract] | 1120019 |
| 6 | PTSD[Title/Abstract] OR posttrauma*[Title/Abstract] OR "post-trauma*"[Title/Abstract] OR "post trauma*"[Title/Abstract] | 97457 |
| 7 | "stress disorder*"[Title/Abstract] | 45815 |
| 8 | OCD[Title/Abstract] OR "obsessive compulsive"[Title/Abstract] OR "obsessive-compulsive"[Title/Abstract] | 25292 |
| 9 | acrophob*[Title/Abstract] OR agoraphob*[Title/Abstract] OR agrophobi*[Title/Abstract] OR claustrophob*[Title/Abstract] OR emetophob*[Title/Abstract] OR arachnophob*[Title/Abstract] OR hypochondri*[Title/Abstract] OR kinesiophob*[Title/Abstract] OR phobi*[Title/Abstract] OR shyness[Title/Abstract] | 25009 |
| 10 | "panic attack*"[Title/Abstract] OR "panic disorder*"[Title/Abstract] OR "panic fear"[Title/Abstract] OR "panic symptom*"[Title/Abstract] | 12944 |
| 11 | #4 OR #5 OR #6 OR #7 OR #8 OR #9 OR #10 | 1308600 |
| 12 | "standardised diagnostic assessment*"[Title/Abstract] OR "standardised diagnostic interview*"[Title/Abstract] | 47 |
| 13 | "standardized diagnostic assessment*"[Title/Abstract] OR "standardized diagnostic interview*"[Title/Abstract] | 188 |
| 14 | "standardised assessment*"[Title/Abstract] OR "standardized assessment*"[Title/Abstract] | 4644 |
| 15 | "computer assisted diagnos*"[Title/Abstract] OR "computer-assisted diagnos*"[Title/Abstract] | 1115 |
| 16 | "diagnostic interview schedule for children"[Title/Abstract] | 478 |
| 17 | DAWBA[Title/Abstract] OR "development and wellbeing assessment*"[Title/Abstract] OR "development and well-being assessment*"[Title/Abstract] | 256 |
| 18 | CAPA[Title/Abstract] OR "child and adolescent psychiatric assessment*"[Title/Abstract] | 896 |
| 19 | K-SADS[Title/Abstract] OR "kiddie schedule for affective disorders and schizophrenia"[Title/Abstract] OR "schedule for affective disorders and schizophrenia for school-age children"[Title/Abstract] | 1038 |
| 20 | BCFPI[Title/Abstract] OR "brief child and family phone interview*"[Title/Abstract] | 9 |
| 21 | #12 OR #13 OR #14 OR #15 OR #16 OR #17 OR #18 OR #19 OR #20 | 8627 |
| 22 | #3 AND #11 AND #21 | 1546 |
| 23 | "Mental Health Services"[Mesh] OR "Child Health Services"[Mesh] OR "Hospitals, Psychiatric"[Mesh] | 156453 |
| 24 | "mental health service*"[Title/Abstract] OR "child* health service*"[Title/Abstract] OR "adolescen* health service*"[Title/Abstract] | 34646 |
| 25 | "child and adolescent mental health service*"[Title/Abstract] OR "child or adolescent mental health service*"[Title/Abstract] OR CAMHS[Title/Abstract] | 1250 |
| 26 | "mental health clinic*"[Title/Abstract] OR "mental health cent*"[Title/Abstract] | 6548 |
| 27 | "clinical practice*"[Title/Abstract] | 296476 |
| 28 | "psychiatric care"[Title/Abstract] OR "psychiatric unit*"[Title/Abstract] OR "psychiatric hospital*"[Title/Abstract] OR "psychiatric service*"[Title/Abstract] OR "psychiatric facilit*"[Title/Abstract] OR "psychiatric institution*"[Title/Abstract] OR "psychiatric setting*"[Title/Abstract] OR "psychiatric system*"[Title/Abstract] OR "psychiatric ward*"[Title/Abstract] OR "psychiatric environment*"[Title/Abstract] | 33846 |
| 29 | "mental health care"[Title/Abstract] OR "mental health unit*"[Title/Abstract] OR "mental health hospital*"[Title/Abstract] OR "mental health facilit*"[Title/Abstract] OR "mental health institution*"[Title/Abstract] OR "mental health setting*"[Title/Abstract] OR "mental health system*"[Title/Abstract] OR "mental health ward*"[Title/Abstract] OR "mental health environment*"[Title/Abstract] | 24291 |
| 30 | "acute care"[Title/Abstract] OR "acute unit*"[Title/Abstract] OR "acute hospital*"[Title/Abstract] OR "acute service*"[Title/Abstract] OR "acute facilit*"[Title/Abstract] OR "acute institution*"[Title/Abstract] OR "acute setting*"[Title/Abstract] OR "acute system*"[Title/Abstract] OR "acute ward*"[Title/Abstract] OR "acute environment*"[Title/Abstract] | 44608 |
| 31 | #23 OR #24 OR #25 OR #26 OR #27 OR #28 OR #29 OR #30 | 543163 |
| 32 | #22 AND #31 | 197 |
| 33 | #22 AND #31  Filters: from 2013 – 2025 | **109** |
| **Platform and database**: Ovid Embase <1974 to 2025 January 31> | | |
| **Link to run search:** <https://access.ovid.com/custom/redirector/wayfless.html?idp=https://idp.eng.nhs.uk/openathens&url=http://ovidsp.ovid.com/ovidweb.cgi?T=JS&NEWS=N&PAGE=main&SHAREDSEARCHID=3Pvy5Gghcq5JDW5Gh6vmA0qM2KlM5FuoYWkTqLgTDetZmv6xN2Ifn4OaVX5AjlXZc> | | |
| 1 | exp Juvenile/ or exp Child/ or exp Infant/ or exp Adolescence/ or exp Adolescent/ or exp Pediatrics/ | 4364684 |
| 2 | (adolescen* or child* or infant* or juvenile* or minor* or paediatric* or pediatric* or teen* or "young person*" or "young people" or youth*).ti,ab. | 3724722 |
| 3 | or/1-2 | 5597652 |
| 4 | exp Anxiety/ or exp Anxiety Disorder/ or exp Depression/ or exp Hypochondriasis/ or exp Dysthymia/ or exp Body Dysmorphic Disorder/ or exp Posttraumatic Stress Disorder/ or exp Agoraphobia/ or exp Social Phobia/ or exp Obsessive Compulsive Disorder/ or exp Mood Disorder/ | 1122738 |
| 5 | (agitat* or anger or anxiet* or anxious or "body dysmorphi*" or compulsion* or depress* or distress or dysthymia or "dysthymic disorder*" or flashback* or GAD or hypervigilance or insomnia or irritable or "lack of motivation" or "low mood" or "low motivation" or "low self-esteem" or "low self esteem" or "mood affective disorder*" or "mood disorder*" or "mood swing*" or nightmare* or panic or phobi* or "self-harm" or "self harm" or "selfharm" or "self-injur*" or "self injur*" or suicid* or tired* or withdrawn).ti,ab. | 1478709 |
| 6 | (PTSD or posttrauma* or "post-trauma*" or "post trauma*").ti,ab. | 117525 |
| 7 | (stress adj disorder*).ti,ab. | 52912 |
| 8 | (OCD or "obsessive compulsive" or "obsessive-compulsive").ti,ab. | 34074 |
| 9 | (acrophob* or agoraphob* or agrophobi* or claustrophob* or emetophob* or arachnophob* or hypochondri* or kinesiophob* or phobi* or shyness).ti,ab. | 32388 |
| 10 | (panic adj2 (attack* or disorder* or fear or symptom*)).ti,ab. | 11748 |
| 11 | or/4-10 | 1894483 |
| 12 | Diagnostic Interview Schedule/ or "schedule for affective disorders and schizophrenia"/ or *mental disease assessment/ | 4280 |
| 13 | ("standardised diagnostic" adj (assessment* or interview*)).ti,ab. | 55 |
| 14 | ("standardized diagnostic" adj (assessment* or interview*)).ti,ab. | 243 |
| 15 | ("standardised assessment*" or "standardized assessment*").ti,ab. | 6544 |
| 16 | ("computer assisted diagnos*" or "computer-assisted diagnos*").ti,ab. | 988 |
| 17 | "diagnostic interview schedule for children".ti,ab. | 545 |
| 18 | (DAWBA or "development and wellbeing assessment*" or "development and well-being assessment*").ti,ab. | 400 |
| 19 | (CAPA or "child and adolescent psychiatric assessment*").ti,ab. | 1190 |
| 20 | (K-SADS or "kiddie schedule for affective disorders and schizophrenia" or "schedule for affective disorders and schizophrenia for school-age children").ti,ab. | 2187 |
| 21 | (BCFPI or "brief child and family phone interview*").ti,ab. | 9 |
| 22 | or/12-21 | 14867 |
| 23 | 3 and 11 and 22 | 3659 |
| 24 | exp Mental Health Service/ or exp Child Health Care/ or exp Mental Health Center/ or exp Mental Hospital/ or exp Mental Health Care/ | 271362 |
| 25 | ((mental or child* or adolescen*) adj "health service*").ti,ab. | 36394 |
| 26 | ("child and adolescent mental health service*" or "child or adolescent mental health service*" or CAMHS).ti,ab. | 2043 |
| 27 | ("mental health clinic*" or "mental health cent*").ti,ab. | 8406 |
| 28 | "clinical practice*".ti,ab. | 422770 |
| 29 | ((psych or psychiatric or "mental health" or acute) adj2 (care or unit* or hospital* or service* or facilit* or institution* or setting* or system* or ward* or environment*)).ti,ab. | 226087 |
| 30 | (acute adj2 ("mental health" or psych or psychiatric)).ti,ab. | 4811 |
| 31 | or/24-30 | 849343 |
| 32 | 23 and 31 | 491 |
| 33 | limit 32 to yr="2013 -Current" | **323** |
| **Platform and database**: Ovid MEDLINE® ALL <1946 to January 31, 2025> | | |
| **Link to run search:** <https://access.ovid.com/custom/redirector/wayfless.html?idp=https://idp.eng.nhs.uk/openathens&url=http://ovidsp.ovid.com/ovidweb.cgi?T=JS&NEWS=N&PAGE=main&SHAREDSEARCHID=6HrfDRJYiZtphZKGcWKNBTcoLlercEaEaJp3AwVdsXE41LMTWXav6Dbt27gPFw2DL> | | |
| 1 | exp Child/ or exp Minors/ or exp Child, Preschool/ or exp Adolescent/ | 3536572 |
| 2 | (adolescen* or child* or infant* or juvenile* or minor* or paediatric* or pediatric* or teen* or "young person*" or "young people" or youth*).tw,kw,kf. | 3042553 |
| 3 | or/1-2 | 4906471 |
| 4 | exp Anxiety/ or exp Anxiety Disorders/ or exp Depression/ or exp Hypochondriasis/ or exp Dysthymic Disorder/ or exp Stress Disorders, Post-Traumatic/ or exp Agoraphobia/ or exp Phobia, Social/ or exp Obsessive-Compulsive Disorder/ or exp Mood Disorders/ or exp Phobic Disorders/ | 491611 |
| 5 | (agitat* or anger or anxiet* or anxious or "body dysmorphi*" or compulsion* or depress* or distress or dysthymia or "dysthymic disorder*" or flashback* or GAD or hypervigilance or insomnia or irritable or "lack of motivation" or "low mood" or "low motivation" or "low self-esteem" or "low self esteem" or "mood affective disorder*" or "mood disorder*" or "mood swing*" or nightmare* or panic or phobi* or "self-harm" or "self harm" or "selfharm" or "self-injur*" or "self injur*" or suicid* or tired* or withdrawn).tw,kw,kf. | 1121430 |
| 6 | (PTSD or posttrauma* or "post-trauma*" or "post trauma*").tw,kw,kf. | 97630 |
| 7 | (stress adj disorder*).tw,kw,kf. | 45951 |
| 8 | (OCD or "obsessive compulsive" or "obsessive-compulsive").tw,kw,kf. | 25305 |
| 9 | (acrophob* or agoraphob* or agrophobi* or claustrophob* or emetophob* or arachnophob* or hypochondri* or kinesiophob* or phobi* or shyness).tw,kw,kf. | 25023 |
| 10 | (panic adj2 (attack* or disorder* or fear or symptom*)).tw,kw,kf. | 13461 |
| 11 | or/4-10 | 1310166 |
| 12 | ("standardised diagnostic" adj (assessment* or interview*)).tw,kw,kf. | 47 |
| 13 | ("standardized diagnostic" adj (assessment* or interview*)).tw,kw,kf. | 188 |
| 14 | ("standardised assessment*" or "standardized assessment*").tw,kw,kf. | 4663 |
| 15 | ("computer assisted diagnos*" or "computer-assisted diagnos*").tw,kw,kf. | 1118 |
| 16 | "diagnostic interview schedule for children".tw,kw,kf. | 478 |
| 17 | (DAWBA or "development and wellbeing assessment*" or "development and well-being assessment*").tw,kw,kf. | 271 |
| 18 | (CAPA or "child and adolescent psychiatric assessment*").tw,kw,kf. | 989 |
| 19 | (K-SADS or "kiddie schedule for affective disorders and schizophrenia" or "schedule for affective disorders and schizophrenia for school-age children").tw,kw,kf. | 1254 |
| 20 | (BCFPI or "brief child and family phone interview*").tw,kw,kf. | 9 |
| 21 | or/12-20 | 8971 |
| 22 | 3 and 11 and 21 | 1759 |
| 23 | exp Mental Health Services/ or exp Child Health Services/ or exp Hospitals, Psychiatric/ | 156547 |
| 24 | ((mental or child* or adolescen*) adj "health service*").tw,kw,kf. | 36117 |
| 25 | ("child and adolescent mental health service*" or "child or adolescent mental health service*" or CAMHS).tw,kw,kf. | 1273 |
| 26 | ("mental health clinic*" or "mental health cent*").tw,kw,kf. | 6606 |
| 27 | "clinical practice*".tw,kw,kf. | 297170 |
| 28 | ((psychiatric or "mental health" or acute) adj2 (care or unit* or hospital* or service* or facilit* or institution* or setting* or system* or ward* or environment*)).tw,kw,kf. | 169584 |
| 29 | (acute adj2 ("mental health" or psychiatric)).tw,kw,kf. | 3630 |
| 30 | or/23-29 | 586617 |
| 31 | 22 and 30 | 238 |
| 32 | limit 31 to yr="2013 -Current" | **130** |
| **Platform and database**: ProQuest APA PsycInfo® | | |
| S1 | ti,ab(infant* OR teen* OR youth* OR youngster* OR adolescent* OR adolescence OR juvenile* OR (young NEAR/1 (person* OR people)) OR child* OR minor* OR p?ediatric*) OR MAINSUBJECT.EXACT("Pediatrics") | 1133403 |
| S2 | MAINSUBJECT.EXACT("Anxiety Disorders" OR "Anxiety" OR "Depression (Emotion)" OR "Major Depression" OR "Dysthymic Disorder" OR "Phobias" OR "Social Phobia" OR "Obsessive Compulsive Disorder") | 315360 |
| S3 | MAINSUBJECT.EXACT("Body Dysmorphic Disorder" OR "Posttraumatic Stress Disorder" OR "Illness Anxiety Disorder" OR "Agoraphobia" OR "Affective Disorders") | 66878 |
| S4 | TIAB(agitat* or anger or anxiet* or anxious or "body dysmorphi*" or compulsion* or depress* or distress or dysthymia or "dysthymic disorder*" or flashback* or GAD or hypervigilance or insomnia or irritable or "lack of motivation" or "low mood" or "low motivation" or "low self-esteem" or "low self esteem" or "mood affective disorder*" or "mood disorder*" or "mood swing*" or nightmare* or panic or phobi* or "self-harm" or "self harm" or "selfharm" or "self-injur*" or "self injur*" or suicid* or tired* or withdrawn) | 680579 |
| S5 | TIAB(PTSD or posttrauma* or "post-trauma*" or ("post trauma" OR "post traumatic") OR "stress disorder" or "stress disorders" OR OCD or "obsessive compulsive" or "obsessive-compulsive" OR acrophob* or agoraphob* or agrophobi* or claustrophob* or emetophob* or arachnophob* or hypochondri* or kinesiophob* or phobi* or shyness OR panic near/2 (attack* or disorder* or fear or symptom*)) | 114919 |
| S6 | [S2] OR [S3] OR [S4] OR [S5] | 761449 |
| S7 | MAINSUBJECT.EXACT("Diagnostic Interview Schedule" OR "Computer Assisted Diagnosis") OR TIAB(("standardised diagnostic" or "standardized diagnostic") near/1 (assessment* or interview*) OR "standardised assessment*" or "standardized assessment*" OR "computer assisted diagnos*" or "computer-assisted diagnos*") | 5397 |
| S8 | TIAB("diagnostic interview schedule for children" OR DAWBA or "development and wellbeing assessment*" or "development and well-being assessment*" OR CAPA or "child and adolescent psychiatric assessment*" OR K-SADS or "kiddie schedule for affective disorders and schizophrenia" or "schedule for affective disorders and schizophrenia for school-age children" OR BCFPI or "brief child and family phone interview*") | 2082 |
| S9 | [S7] OR [S8] | 7384 |
| S10 | [S1] AND [S6] AND [S9] | 1513 |
| S11 | MAINSUBJECT.EXACT("Clinical Practice" OR "Mental Health Services" OR "Psychiatric Hospitals") OR TIAB((mental or child* or adolescen*) near/1 ("health service" OR "health services")) | 97752 |
| S12 | TIAB("child and adolescent mental health service*" or "child or adolescent mental health service*" or CAMHS OR "mental health clinic*" or "mental health cent*" OR "clinical practice" or "clinical practices") | 64754 |
| S13 | TIAB((psychiatric or "mental health" or acute) near/2 (care or unit* or hospital* or service* or facilit* or institution* or setting* or system* or ward* or environment*) OR acute near/2 ("mental health" or psychiatric)) | 108082 |
| S14 | [S11] or [S12] or [S13] | 203912 |
| S15 | [S10] AND [S14] | 187 |
| S16 | ([S10] AND [S14]) AND pd(20130101-20251231) | **99** |
| **Platform and database**: ProQuest Applied Social Sciences Index & Abstracts (ASSIA) | | |
| S1 | MAINSUBJECT.EXACT.EXPLODE("Adolescents") OR MAINSUBJECT.EXACT.EXPLODE("Children") OR MAINSUBJECT.EXACT.EXPLODE("Infants") | 137188 |
| S2 | title(adolescen* OR child* OR infant* OR juvenile* OR minor* OR paediatric* OR pediatric* OR teen* OR "young person" OR "young persons" OR "young people" OR youth*) OR abstract(adolescen* OR child* OR infant* OR juvenile* OR minor* OR paediatric* OR pediatric* OR teen* OR "young person" OR "young persons" OR "young people" OR youth*) | 335315 |
| S3 | [S1] or [S2] | 342145 |
| S4 | MAINSUBJECT.EXACT.EXPLODE("Hypochondriasis") OR MAINSUBJECT.EXACT.EXPLODE("Anxiety") OR MAINSUBJECT.EXACT.EXPLODE("Anxiety disorders") OR MAINSUBJECT.EXACT.EXPLODE("Body dysmorphic disorder") OR MAINSUBJECT.EXACT.EXPLODE("Fear & phobias") OR MAINSUBJECT.EXACT.EXPLODE("Obsessive-Compulsive neuroses") OR MAINSUBJECT.EXACT.EXPLODE("Agoraphobia") OR MAINSUBJECT.EXACT.EXPLODE("Emotional disorders") OR MAINSUBJECT.EXACT.EXPLODE("Mental depression") OR MAINSUBJECT.EXACT.EXPLODE("Post traumatic stress disorder") | 74511 |
| S5 | title(agitat* or anger or anxiet* or anxious or "body dysmorphic" or compulsion* or depress* or distress or dysthymia or "dysthymic disorder" or flashback* or GAD or hypervigilance or insomnia or irritable or "lack of motivation" or "low mood" or "low motivation" or "low self-esteem" or "low self esteem" or "mood affective disorder*" or "mood disorder" OR "mood disorders" or "mood swing" OR "mood swings" or nightmare* or panic or phobi* or "self-harm" or "self harm" or "selfharm" or "self-injur*" or "self injuring" OR "self injurious" OR "self injury" or suicid* or tired* or withdrawn) OR abstract(agitat* or anger or anxiet* or anxious or "body dysmorphic" or compulsion* or depress* or distress or dysthymia or "dysthymic disorder" or flashback* or GAD or hypervigilance or insomnia or irritable or "lack of motivation" or "low mood" or "low motivation" or "low self-esteem" or "low self esteem" or "mood affective disorder*" or "mood disorder" OR "mood disorders" or "mood swing" OR "mood swings" or nightmare* or panic or phobi* or "self-harm" or "self harm" or "selfharm" or "self-injur*" or "self injuring" OR "self injurious" OR "self injury" or suicid* or tired* or withdrawn) | 155495 |
| S6 | title(PTSD or posttrauma* or "post-trauma*" or "post trauma" OR "post traumatic") OR abstract(PTSD or posttrauma* or "post-trauma*" or "post trauma" OR "post traumatic") | 13413 |
| S7 | title(stress NEAR/1 disorder*) OR abstract(stress NEAR/1 disorder*) | 8984 |
| S8 | title(OCD or "obsessive compulsive" or "obsessive-compulsive") OR abstract(OCD or "obsessive compulsive" or "obsessive-compulsive") | 4031 |
| S9 | title((acrophob* or agoraphob* or agrophobi* or claustrophob* or emetophob* or arachnophob* or hypochondri* or kinesiophob* or phobi* or shyness) ) OR abstract((acrophob* or agoraphob* or agrophobi* or claustrophob* or emetophob* or arachnophob* or hypochondri* or kinesiophob* or phobi* or shyness) ) | 4788 |
| S10 | title(panic NEAR/2 (attack* or disorder* or fear or symptom*) ) OR abstract(panic NEAR/2 (attack* or disorder* or fear or symptom*) ) | 2459 |
| S11 | [S4] or [S5] or [S6] or [S7] or [S8] or [S9] or [S10] | 176353 |
| S12 | [S3] and [S11] | 55759 |
| S13 | title("standardised diagnostic" NEAR/1 (assessment* or interview*)) OR abstract("standardised diagnostic" NEAR/1 (assessment* or interview*)) | 24 |
| S14 | title("standardized diagnostic" NEAR/1 (assessment* or interview*)) OR abstract("standardized diagnostic" NEAR/1 (assessment* or interview*)) | 41 |
| S15 | title("standardised assessment*" or "standardized assessment*") OR abstract("standardised assessment*" or "standardized assessment*") | 791 |
| S16 | title("computer assisted diagnos*" or "computer-assisted diagnos*") OR abstract("computer assisted diagnos*" or "computer-assisted diagnos*") | 5 |
| S17 | title("diagnostic interview schedule for children") OR abstract("diagnostic interview schedule for children") | 287 |
| S18 | title(DAWBA or "development and wellbeing assessment*" or "development and well-being assessment*") OR abstract(DAWBA or "development and wellbeing assessment*" or "development and well-being assessment*") | 117 |
| S19 | title(CAPA or "child and adolescent psychiatric assessment*") OR abstract(CAPA or "child and adolescent psychiatric assessment*") | 80 |
| S20 | title(K-SADS or "kiddie schedule for affective disorders and schizophrenia" or "schedule for affective disorders and schizophrenia for school-age children") OR abstract(K-SADS or "kiddie schedule for affective disorders and schizophrenia" or "schedule for affective disorders and schizophrenia for school-age children") | 338 |
| S21 | title(BCFPI or "brief child and family phone interview*") OR abstract(BCFPI or "brief child and family phone interview*") | 6 |
| S22 | [S13] or [S14] or [S15] or [S16] or [S17] or [S18] or [S19] or [S20] or [S21] | 1658 |
| S23 | [S12] and [S22] | 533 |
| S24 | MAINSUBJECT.EXACT.EXPLODE("Psychiatric hospitals") OR MAINSUBJECT.EXACT.EXPLODE("Mental health services") OR MAINSUBJECT.EXACT.EXPLODE("Mental health care") | 34047 |
| S25 | title((mental or child* or adolescen*) NEAR/1 ("health service" OR "health services")) OR abstract((mental or child* or adolescen*) NEAR/1 ("health service" OR "health services")) | 13955 |
| S26 | title("child and adolescent mental health service*" or "child or adolescent mental health service*" or CAMHS) OR abstract("child and adolescent mental health service*" or "child or adolescent mental health service*" or CAMHS) | 790 |
| S27 | title("mental health clinic*" or "mental health cent*") OR abstract("mental health clinic*" or "mental health cent*") | 2193 |
| S28 | title("clinical practice" OR "clinical practices") OR abstract("clinical practice" OR "clinical practices") | 17017 |
| S29 | title((psych or psychiatric or "mental health" or acute) NEAR/2 (care or unit* or hospital* or service* or facilit* or institution* or setting* or system* or ward* or environment*)) OR abstract((psych or psychiatric or "mental health" or acute) NEAR/2 (care or unit* or hospital* or service* or facilit* or institution* or setting* or system* or ward* or environment*)) | 39128 |
| S30 | title(acute NEAR/2 ("mental health" or psych or psychiatric) ) OR abstract(acute NEAR/2 ("mental health" or psych or psychiatric) ) | 1294 |
| S31 | [S24] or [S25] or [S26] or [S27] or [S28] or [S29] or [S30] | 72901 |
| S32 | [S23] and [S31] | 93 |
| S33 | ([S23] and [S31]) AND pd(20130101-20251231) | **50** |
| **Platform and database**: ProQuest International Bibliography of the Social Sciences (IBSS) | | |
| S1 | MAINSUBJECT.EXACT.EXPLODE("Adolescents") OR MAINSUBJECT.EXACT.EXPLODE("Children") OR MAINSUBJECT.EXACT.EXPLODE("Pediatrics") | 86522 |
| S2 | title(adolescen* OR child* OR infant* OR juvenile* OR minor* OR paediatric* OR pediatric* OR teen* OR ("young person" OR "young persons") OR "young people" OR youth*) OR abstract(adolescen* OR child* OR infant* OR juvenile* OR minor* OR paediatric* OR pediatric* OR teen* OR ("young person" OR "young persons") OR "young people" OR youth*) | 307281 |
| S3 | [S1] or [S2] | 315656 |
| S4 | MAINSUBJECT.EXACT("Hypochondriasis") OR MAINSUBJECT.EXACT.EXPLODE("Anxiety") OR MAINSUBJECT.EXACT.EXPLODE("Anxiety disorders") OR MAINSUBJECT.EXACT.EXPLODE("Obsessive-Compulsive neuroses") OR MAINSUBJECT.EXACT.EXPLODE("Body dysmorphic disorder") OR MAINSUBJECT.EXACT.EXPLODE("Fear & phobias") OR MAINSUBJECT.EXACT.EXPLODE("Mental depression") OR MAINSUBJECT.EXACT.EXPLODE("Emotional disorders") OR MAINSUBJECT.EXACT.EXPLODE("Post traumatic stress disorder") | 33139 |
| S5 | title(agitat* or anger or anxiet* or anxious or ("body dysmorphic") or compulsion* or depress* or distress or dysthymia or ("dysthymic disorder") or flashback* or GAD or hypervigilance or insomnia or irritable or "lack of motivation" or "low mood" or "low motivation" or "low self-esteem" or "low self esteem" or "mood affective disorder*" or ("mood disorder" OR "mood disorders") or ("mood swing" OR "mood swings") or nightmare* or panic or phobi* or "self-harm" or "self harm" or "selfharm" or "self-injur*" or ("self injuring" OR "self injurious" OR "self injury") or suicid* or tired* or withdrawn) OR abstract(agitat* or anger or anxiet* or anxious or ("body dysmorphic") or compulsion* or depress* or distress or dysthymia or ("dysthymic disorder") or flashback* or GAD or hypervigilance or insomnia or irritable or "lack of motivation" or "low mood" or "low motivation" or "low self-esteem" or "low self esteem" or "mood affective disorder*" or ("mood disorder" OR "mood disorders") or ("mood swing" OR "mood swings") or nightmare* or panic or phobi* or "self-harm" or "self harm" or "selfharm" or "self-injur*" or ("self injuring" OR "self injurious" OR "self injury") or suicid* or tired* or withdrawn) | 80491 |
| S6 | title(PTSD or posttrauma* or "post-trauma*" or "post trauma" OR "post traumatic") OR abstract(PTSD or posttrauma* or "post-trauma*" or "post trauma" OR "post traumatic") | 3607 |
| S7 | title((stress NEAR/1 disorder*)) OR abstract((stress NEAR/1 disorder*)) | 2266 |
| S8 | title(OCD or "obsessive compulsive" or "obsessive-compulsive") OR abstract(OCD or "obsessive compulsive" or "obsessive-compulsive") | 427 |
| S9 | title(acrophob* or agoraphob* or agrophobi* or claustrophob* or emetophob* or arachnophob* or hypochondri* or kinesiophob* or phobi* or shyness) OR abstract(acrophob* or agoraphob* or agrophobi* or claustrophob* or emetophob* or arachnophob* or hypochondri* or kinesiophob* or phobi* or shyness) | 1337 |
| S10 | title(panic NEAR/2 (attack* or disorder* or fear or symptom*)) OR abstract(panic NEAR/2 (attack* or disorder* or fear or symptom*)) | 447 |
| S11 | [S4] or [S5] or [S6] or [S7] or [S8] or [S9] or [S10] | 95053 |
| S12 | [S3] and [S11] | 19057 |
| S13 | title(("standardised diagnostic" NEAR/1 (assessment* or interview*)) ) OR abstract(("standardised diagnostic" NEAR/1 (assessment* or interview*)) ) | 0 |
| S14 | title(("standardized diagnostic" NEAR/1 (assessment* or interview*)) ) OR abstract(("standardized diagnostic" NEAR/1 (assessment* or interview*)) ) | 6 |
| S15 | title("standardised assessment*" or "standardized assessment*") OR abstract("standardised assessment*" or "standardized assessment*") | 167 |
| S16 | title("computer assisted diagnos*" or "computer-assisted diagnos*") OR abstract("computer assisted diagnos*" or "computer-assisted diagnos*") | 1 |
| S17 | title("diagnostic interview schedule for children") OR abstract("diagnostic interview schedule for children") | 27 |
| S18 | title(DAWBA or "development and wellbeing assessment*" or "development and well-being assessment*") OR abstract(DAWBA or "development and wellbeing assessment*" or "development and well-being assessment*") | 1 |
| S19 | title(CAPA or "child and adolescent psychiatric assessment*") OR abstract(CAPA or "child and adolescent psychiatric assessment*") | 84 |
| S20 | title(K-SADS or "kiddie schedule for affective disorders and schizophrenia" or "schedule for affective disorders and schizophrenia for school-age children") OR abstract(K-SADS or "kiddie schedule for affective disorders and schizophrenia" or "schedule for affective disorders and schizophrenia for school-age children") | 15 |
| S21 | title(BCFPI or "brief child and family phone interview*") OR abstract(BCFPI or "brief child and family phone interview*") | 0 |
| S22 | [S13] or [S14] or [S15] or [S16] or [S17] or [S18] or [S19] or [S20] or [S21] | 301 |
| S23 | [S12] and [S22] | 35 |
| S24 | ([S12] and [S22]) AND pd(20130101-20251231) | **16** |
| **Platform and database**: Cochrane Library | | |
| **Link to run search:** <https://www.cochranelibrary.com/advanced-search/search-manager?search=7638315> | | |
| #1 | MeSH descriptor: [Child] explode all trees | 83857 |
| #2 | [mh minors] | 15 |
| #3 | [mh "child, preschool"] | 40450 |
| #4 | [mh adolescent] | 139606 |
| #5 | [mh pediatrics] | 1060 |
| #6 | (adolescen* or child* or infant* or juvenile* or minor* or paediatric* or pediatric* or teen* or young NEXT person* or "young people" or youth*):ti,ab,kw | 399948 |
| #7 | #1 or #2 or #3 or #4 or #5 or #6 | 399977 |
| #8 | [mh anxiety] | 13320 |
| #9 | [mh "anxiety disorders"] | 10619 |
| #10 | [mh depression] | 19070 |
| #11 | [mh hypochondriasis] | 105 |
| #12 | [mh "Dysthymic Disorder"] | 203 |
| #13 | [mh "Stress Disorders, Post-Traumatic"] | 4293 |
| #14 | [mh agoraphobia] | 503 |
| #15 | [mh "phobia, social"] | 436 |
| #16 | [mh "Obsessive-Compulsive Disorder"] | 1570 |
| #17 | [mh "mood disorders"] | 20745 |
| #18 | [mh "phobic disorders"] | 2212 |
| #19 | (agitat* or anger or anxiet* or anxious or body next dysmorphi* or compulsion* or depress* or distress or dysthymia or dysthymic next disorder* or flashback* or GAD or hypervigilance or insomnia or irritable or "lack of motivation" or "low mood" or "low motivation" or "low self-esteem" or "low self esteem" or mood next affective next disorder* or mood next disorder* or mood next swing* or nightmare* or panic or phobi* or "self-harm" or "self harm" or "selfharm" or self next injur* or suicid* or tired* or withdrawn):ti,ab,kw | 221532 |
| #20 | (PTSD or posttrauma* or post next trauma* or "post-trauma"):ti,ab,kw | 11914 |
| #21 | (stress NEAR/1 disorder*):ti,ab,kw | 8724 |
| #22 | (OCD or "obsessive compulsive" or "obsessive-compulsive"):ti,ab,kw | 4244 |
| #23 | (acrophob* or agoraphob* or agrophobi* or claustrophob* or emetophob* or arachnophob* or hypochondri* or kinesiophob* or phobi* or shyness):ti,ab,kw | 7105 |
| #24 | (panic NEAR/2 (attack* or disorder* or fear or symptom*)):ti,ab,kw | 2873 |
| #25 | #8 OR #9 OR #10 OR #11 OR #12 OR #13 OR #14 OR #15 OR #16 OR #17 OR #18 OR #19 OR #20 OR #21 OR #22 OR #23 OR #24 | 232141 |
| #26 | ("standardised diagnostic" NEAR/1 (assessment* or interview*)):ti,ab,kw | 0 |
| #27 | ("standardized diagnostic" NEAR/1 (assessment* or interview*)):ti,ab,kw | 0 |
| #28 | ("computer assisted diagnosis" or "computer assisted diagnoses"):ti,ab,kw | 172 |
| #29 | (standardised next assessment* or standardized next assessment*):ti,ab,kw | 648 |
| #30 | ("diagnostic interview schedule for children"):ti,ab,kw | 47 |
| #31 | (DAWBA or "development and wellbeing assessment" or "development and well being assessment"):ti,ab,kw | 30 |
| #32 | (CAPA or "child and adolescent psychiatric assessment"):ti,ab,kw | 57 |
| #33 | (K-SADS or "kiddie schedule for affective disorders and schizophrenia" or "schedule for affective disorders and schizophrenia for school age children"):ti,ab,kw | 247 |
| #34 | (BCFPI or "brief child and family phone interview"):ti,ab,kw | 2 |
| #35 | #26 OR #27 OR #28 OR #29 OR #30 OR #31 OR #32 OR #33 OR #34 | 1197 |
| #36 | #7 AND #25 AND #35 | 254 |
| #37 | [mh "mental health services"] | 9706 |
| #38 | [mh "child health services"] | 1294 |
| #39 | [mh "Hospitals, Psychiatric"] | 307 |
| #40 | ((mental or child* or adolescen*) NEAR/1 health next service*):ti,ab,kw | 5458 |
| #41 | ("child and adolescent mental health service" or "child and adolescent mental health services" or "child or adolescent mental health service" or "child or adolescent mental health services" or CAMHS):ti,ab,kw | 189 |
| #42 | ("mental health clinic" or "mental health clinics" or "mental health center" or "mental health centre" or "mental health centers" or "mental health centres"):ti,ab,kw | 1115 |
| #43 | ((psychiatric or "mental health" or acute) NEAR/2 (care or unit* or hospital* or service* or facilit* or institution* or setting* or system* or ward* or environment*)):ti,ab,kw | 19281 |
| #44 | (acute NEAR/2 ("mental health" or psychiatric)):ti,ab,kw | 383 |
| #45 | #37 OR #38 OR #39 OR #40 OR #41 OR #42 OR #43 OR #44 | 29059 |
| #46 | #36 AND #45 | 46 |
| #47 | Custom Range: 2013 to 2025 | **29** |
| **Platform and database**: Database of Abstracts of Reviews of Effects (DARE) | | |
|  | (adolescen* or child* or infant* or juvenile* or minor* or paediatric* or pediatric* or teen* or young person* or young people or youth*) |  |
|  | AND |  |
|  | (agitat* or anger or anxiet* or anxious or body dysmorphi* or compulsion* or depress* or distress or dysthymia or dysthymic disorder* or flashback* or GAD or hypervigilance or insomnia or irritable or lack of motivation or low mood or low motivation or low self-esteem or low self esteem or mood affective disorder* or mood disorder* or mood swing* or nightmare* or panic or phobi* or self-harm or self harm or selfharm or self-injur* or self injur* or suicid* or tired* or withdrawn or PTSD OR posttrauma* OR post-trauma* OR post trauma* OR OCD OR obsessive compulsive OR obsessive-compulsive OR phobi* OR panic) |  |
|  | AND |  |
|  | (standardised diagnostic OR standardized diagnostic OR standardised assessment* OR standardized assessment* OR diagnostic interview OR development and wellbeing assessment OR child and adolescent psychiatric assessment* OR kiddie schedule OR schedule for affective disorders OR brief child and family phone interview*) |  |
|  | Limit 2013 to date | **0** |
